# Q&A: Cognitive ethology - inside the minds of other species

**DOI:** 10.1186/1741-7007-11-108

**Published:** 2013-10-31

**Authors:** Drew Rendall

**Affiliations:** 1 Behavior and Evolution Research Group, Department of Psychology, The University of Lethbridge, Lethbridge, Alberta T1K 3M4, Canada

## Animal cognition is a hot topic. How do we know what other animals are thinking?

Well, actually, mostly we don’t. It’s still a young field and a tricky one. We often can’t be sure even what other people are thinking, even though we can talk to them directly and share the same language. ('Yuh, sure she *said* that, but did she really *mean* it? What is she *actually* trying to *tell* me?’) And animals don’t talk, at least in the usual sense, which makes it even tougher to know what they’re thinking. Furthermore, many animal species live very different lives from us, and so, as the philosopher Wittgenstein famously cautioned, even if they could talk, we probably would not understand what they had to say.

**Figure 1:**
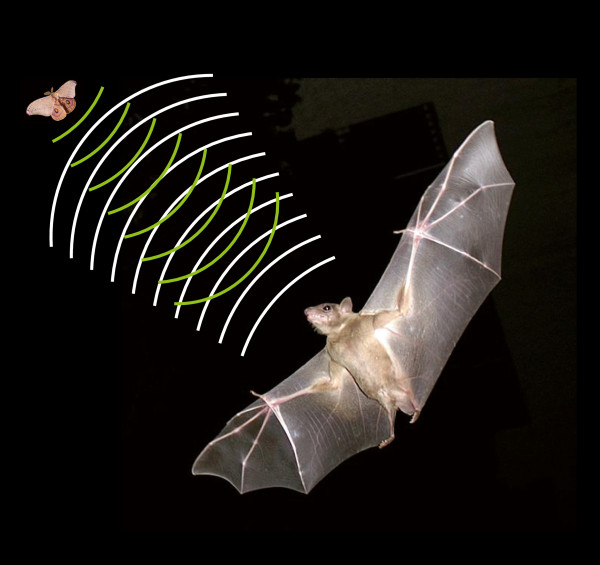
Bats navigate and identify prey by echolocation.

Nevertheless, trying to understand the mental lives of other species - to get inside their heads - is an active and exciting area of research, and, notwithstanding the above caveats, a lot of it, in fact, focuses on aspects of communication, sparked by the example of research on bat echolocation by Donald Griffin and others in the 1950s. They discovered how it is that bats can navigate in the dark, and not by special powers of vision, but by sound - by producing a continuous stream of high-frequency 'click’ sounds (that we humans can’t hear) while they fly (Figure 1) and then listening for the portions of these clicks that get reflected (echoed) back off obstacles in the environment. This work helped to illuminate the dark world of the bat and it catalyzed the modern era of research on animal thinking - what is often now labeled 'cognitive ethology’. Following Griffin’s lead [1], one entire branch of this field focuses on studying the communication systems of other species, believing as he did that these offer a privileged window into the workings of their minds.

## So what have studies of communication told us about the minds of other species?

Well, as the example of bat echolocation itself showed, some other species are likely to have mental lives very different from ours. They see the world very differently and probably think very differently about it as a consequence. Bats effectively 'see’ in sound (although they can also see) and they navigate in the dark by 'talking to themselves’. Exactly what kinds of thoughts they might have about the world remains an open question, but, given their very different experience of the world, whatever thoughts they have (if any) are likely to be quite different, possibly almost unimaginable to us as a grounded, visually dominated species [2,3].

## What about animals more like us? Aren’t they more likely to see the world and think about it similarly to the ways we do?

That’s certainly a sensible intuition that is partially supported. For example, another landmark study in cognitive ethology concerns research on vervet monkeys, which are small African primates. These monkeys are vulnerable to a variety of predators, including leopards, eagles, and large snakes, and studies in the 1970s and 80s found that the monkeys produced different alarm vocalizations in response to each [4]. When other vervets heard the alarm calls of their companions, they automatically engaged escape responses that were different and appropriate for evading a leopard, an eagle or a snake even if these listeners had not themselves yet seen and identified the particular predator involved. It was as though the alarm calls on their own told others everything they needed to know, serving in effect as symbolic labels for the different predators much like our own human words for 'leopard’ , 'eagle’ and 'snake’. So, here was a case where a species more closely related to humans seemed to see the world and to communicate about it much as we do. This was exciting not just for suggesting that the capacity for symbolic communication and thought might exist in a species other than our own but also for what this fact suggested about the deep history of complex thought and language in humans. In other words, it might help us to understand another species better, and it might also help us to understand ourselves better.

## So what do vervet alarm calls actually tell us about the deep history of human thought and language?

Well, the evolutionary origins of complex language and cognition in the human lineage are another hot, and hotly debated, topic, susceptible to many of the same problems that plague studies of animal thinking and more [5]. It’s hard enough to ascertain what others around you are thinking, whether animal or human. It’s harder still when your subjects are fossil species long extinct. How do you reconstruct the thoughts of long dead ancestors? Here, the vervet alarm call story offered a promising lead. Contrary to some evolutionary scenarios that assume a very recent origin of symbolic thought, language and culture in humans, the evidence for symbolic communication in vervet monkeys - with whom we share only a very distant primate ancestor - suggested that the cognitive foundations for these abilities might be very deep indeed, long preceding our transition from monkey-like primates to human ones.

## Does this mean human language and cognition are not as special as we often assume?

Not necessarily. Accumulating evidence since the original vervet work now points to some fundamental differences in the capacity for symbolic thought and communication in nonhuman primates compared to humans. For example, more recent studies of vervet alarm calls suggest that important details of the original work need to be revised and that the symbolic capacity of the monkeys’ calls may be fundamentally different from that of human words. These newer findings align with a large body of other research comparing the cognitive underpinnings of human language and primate communication [6].

In humans, language use is fundamentally intentional, in two senses. It is intentional in the formal philosophical sense that language users make implicit assumptions that the other people they interact with are mental agents, like themselves, with thoughts and beliefs about the world. It is also intentional in the more colloquial sense that speakers deliberately mean, or intend, to share what they know about the world, and influence what others think and know about it, through the use of words that codify thoughts and beliefs. In contrast, intentionality in both senses is largely absent in nonhuman primates, with the possible exception of some of the great apes [7]. In general, primates do not understand each other as mental agents with thoughts and beliefs, or knowledge about the world. Nor do they communicate with the intent of sharing or providing information about the world to others. In short, they seem not to know what others know about the world, or even that others might know things about the world at all. They therefore also fail to appreciate that what others know can be affected by one’s own communications [8]. So, in a fascinating paradox, the alarm calls that vervet monkeys produce function to alert, or inform, others about the presence of dangerous predators, but the effect is inadvertent or unwitting. The monkeys themselves do not understand the effect their calls have.

## So are there other important differences between human language and primate communication?

**Figure 2:**
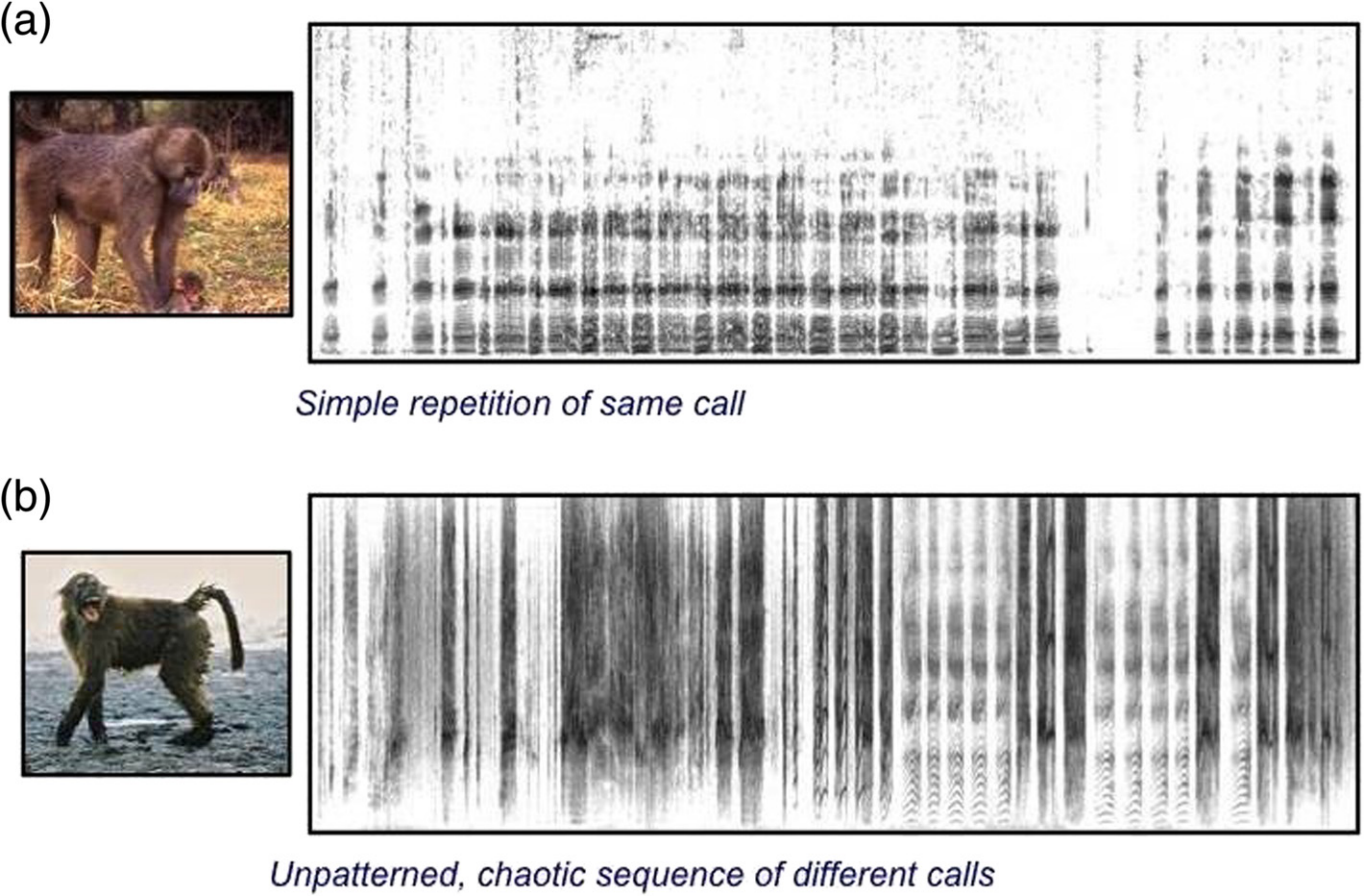
**Nonhuman primate vocal repertoires typically contain a relatively small number of different call types and little evidence of syntax. (a)** The right-hand panel shows a spectrogram of a long sequence of 'grunt’ vocalizations produced by an adult male baboon while approaching and attempting to handle a young infant. Although this is a long sequence of vocalizations, there is no variation in the calls produced throughout the bout. Rather, the same call is simply repeated over and over many times. **(b)** A spectrogram of a long bout of distress calls produced by a juvenile baboon that has been forcibly ejected from a feeding site. In this case, the young baboon produces a long stream of broad-band, noisy vocalizations with some more harmonically structured vocalizations interspersed towards the end of the bout. The vocalizations in this bout exhibit tremendous structural variety, they are not simply a single type of call repeated over and over. However, the sequence is notable for being completely unpatterned, or chaotic, and thus evincing no evidence of syntax, which nevertheless may be functional in the contexts in which these calls are produced [9,10].

Yes, there are. Human languages are organized hierarchically, with short and relatively meaningless sound units (phonemes) combined to form longer meaningful (symbolic) words that are strung together into even longer meaning-bearing phrases and sentences, and, of course, words, phrases and sentences can be recombined endlessly to create an infinite number of different messages. In contrast, closely related nonhuman primates have relatively small signal repertoires with specific vocalizations produced singly or in short bouts without obvious patterning or sequencing that affects the messages produced (Figure 2).

## Does this mean primates are a lot simpler than we tend to think?

Possibly, but not necessarily. It might mean only that the signals they use are simpler and less diverse because they serve relatively broad social functions. Many primates are intensely social. Who you are and what your relationships are to others in the group are critical, and the importance of particular relationships can vary depending on the social context. In this kind of setting, the messages conveyed by vocalizations might be relatively straightforward, perhaps simply announcing your identity - in effect, simply reminding others who you are [11]. But complexity might then arise in the variety of responses that can flow from such announcements for others based on their relationship to you and to others in the group and how those relationships are best prioritized in the particular social context involved. This represents a complex social calculus for which primates appear to be especially adapted. So, it’s possible that the relatively sophisticated social-inferential abilities of primates have effectively relieved signalers from having to develop a larger, more complex repertoire of signals to convey many different messages. Perhaps for primates, the onus of communication is on listeners more than on signalers. Primates do, after all, have large brains and we have to assume all that brain tissue is there for a reason and doing something. Still, it remains an important puzzle to solve.

## Is language unique then in being hierarchically organized and having complex recombinatorial properties?

**Figure 3:**
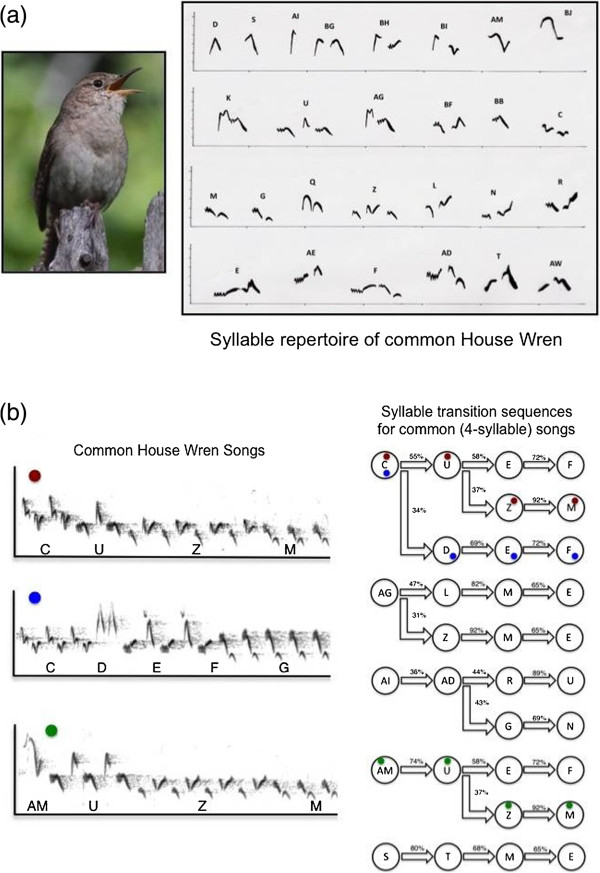
**The signal repertoire of the house wren.** In contrast to the signals of the baboons illustrated in Figure 2, the signal repertoire of the house wren (*Troglodytes aedon*) **(a, left panel)**, a common songbird, is diverse and highly structured [15]. In this example, from a population from Western Canada, the repertoire consists of 27 different types of syllable **(a, right panel)**, where each syllable type is composed of one, two, or in some cases three different individual notes. These syllables are then strung together in longer songs that involve the concatenation of multiple different syllable types. Within a song, each syllable type can be repeated several times before switching to the next syllable type. Further, there are consistent patterns in which syllable types co-occur within songs and in what order they typically appear. **(b)** Templates (right panel) for the 10 most common song types for Western Canadian wrens, three of which are illustrated in the spectrograms in the left panel. The values between syllable types within each song correspond to the transition probabilities between successive syllable types in a song. Regularities in the syllable constitution and syllable transition patterns of song types constitutes a rudimentary syntax.

Not exactly, because, although most primates do not manifest these characteristics, they are present in the communications of an entirely different group of animals, evolutionarily quite remote from humans, namely birds. Birdsong has long been noted for its structural complexity (and resulting aesthetic beauty) and recombinatorial properties. In fact, there are a number of instructive parallels to language. For example, bird song is also hierarchically organized, composed of a repertoire of different notes that are joined in different combinations into discrete syllables that, in turn, are combined in regular patterns, sometimes called phrases or motifs, which themselves can be mixed and sequenced into long song themes in rule-governed ways reminiscent of the grammatical or syntactical rules that structure the complex statements of language (Figure 3) [12,13]. The paradox of birdsong is that its tremendous structural variety and grammatical sequencing do not seem to support any great variety of messages. In general, birdsong serves two major functions: attracting mates and repelling rivals. And both functions may be served by a common message of, once again, simply announcing identity, but doing so in diverse ways that reveal additional characteristics of the signaler that are relevant to mates and rivals. There is some suggestion that songs can differ in different contexts in ways that might support some diversification of messages [14], but, in general, the structural complexity and recombinatorial properties of birdsong appear not to yield a large number of different messages in the same way these characteristics do in human language. And why this might be the case is another outstanding puzzle to be solved.

## What’s the most important lesson to draw from this history of research?

Arguably, the most important lesson is that the evolutionary process can yield a complex and sometimes surprising mosaic of outcomes. As the examples of communication and cognition in humans, primates and songbirds nicely illustrate, closely related species with more similar histories (human and nonhuman primates) are often similar in many respects. But they can also be very different. The psychological intentionality that undergirds complex language in humans, and distinguishes it from the communications of nonhuman primates, also supports a variety of other complex human traits, including our capacity for pedagogy, for empathy, and for broad-scale cooperation, all of which might also prove unique to humans [6]. So, it’s a mental difference that makes a difference. At the same time, very distantly related species, with divergent evolutionary histories (humans and songbirds), are usually quite different but they can be surprisingly similar in some respects. There is nothing yet in the corpus of general evolutionary principles that would lead us to expect in advance that the communication systems of songbirds would have so much in common with human language. Why there is such communicative convergence between songbirds and humans remains an important puzzle to be solved, but one thing we know is that it has also produced some interesting convergences in the way bird and human brains are structured and organized (despite their vast differences in size).

Ultimately, both the similarities among species and their differences are equally informative in our efforts to understand the organization and evolution of various kinds of minds. But the mosaic pattern of outcomes we’ve seen so far certainly reminds us that there can be a host of different solutions to life’s common problems and therefore myriad evolutionary paths species can take. In exploring these possible paths, it’s important that we, as researchers, not pre-judge the places they are likely to lead, or allow our human biases and expectations to color what we think we see in other species. We may be in for a lot of surprises yet.
